# Morphogenetic processes in the development and evolution of the arteries of the pharyngeal arches: their relations to congenital cardiovascular malformations

**DOI:** 10.3389/fcell.2023.1259175

**Published:** 2023-10-12

**Authors:** Anthony Graham, Jill P. J. M. Hikspoors, Wouter H. Lamers, Robert H. Anderson, Simon D. Bamforth

**Affiliations:** ^1^ Centre for Developmental Neurobiology, King’s College London, London, United Kingdom; ^2^ Department of Anatomy and Embryology, Maastricht University, Maastricht, Netherlands; ^3^ Centre for Life, Faculty of Medical Sciences, Biosciences Institute, Newcastle University, Newcastle upon Tyne, United Kingdom

**Keywords:** pharyngeal arch arteries, aortic arch arteries, aorta, arterial duct, fifth arch artery

## Abstract

The heart and aortic arch arteries in amniotes form a double circulation, taking oxygenated blood from the heart to the body and deoxygenated blood to the lungs. These major vessels are formed in embryonic development from a series of paired and symmetrical arteries that undergo a complex remodelling process to form the asymmetric arch arteries in the adult. These embryonic arteries form in the pharyngeal arches, which are symmetrical bulges on the lateral surface of the head. The pharyngeal arches, and their associated arteries, are found in all classes of vertebrates, but the number varies, typically with the number of arches reducing through evolution. For example, jawed vertebrates have six pairs of pharyngeal arch arteries but amniotes, a clade of tetrapod vertebrates, have five pairs. This had led to the unusual numbering system attributed to each of the pharyngeal arch arteries in amniotes (1, 2, 3, 4, and 6). We, therefore, propose that these instead be given names to reflect the vessel: mandibular (1^st^), hyoid (2^nd^), carotid (3^rd^), aortic (4^th^) and pulmonary (most caudal). Aberrant arch artery formation or remodelling leads to life-threatening congenital cardiovascular malformations, such as interruption of the aortic arch, cervical origin of arteries, and vascular rings. We discuss why an alleged fifth arch artery has erroneously been used to interpret congenital cardiac lesions, which are better explained as abnormal collateral channels, or remodelling of the aortic sac.

## Introduction

Congenital cardiovascular malformations are a major cause of death and morbidity from birth, affecting up to 1% of the population. Severe defects involving the morphogenesis of the pharyngeal arch arteries, for example, interruption of the aortic arch, prevent oxygenated blood from the heart being adequately delivered to the body. The asymmetrical arrangement of the arch arteries found in postnatal life requires major remodelling of the symmetrical embryonic precursor blood vessels called the pharyngeal arch arteries. These vessels form within the transient structures known as the pharyngeal arches. In this review, we discuss this remodelling in light of the known evolutionary trends, showing how the changes are related to congenital cardiovascular malformations.

## The pharyngeal arches in mammalian embryos

**FIGURE 1:**
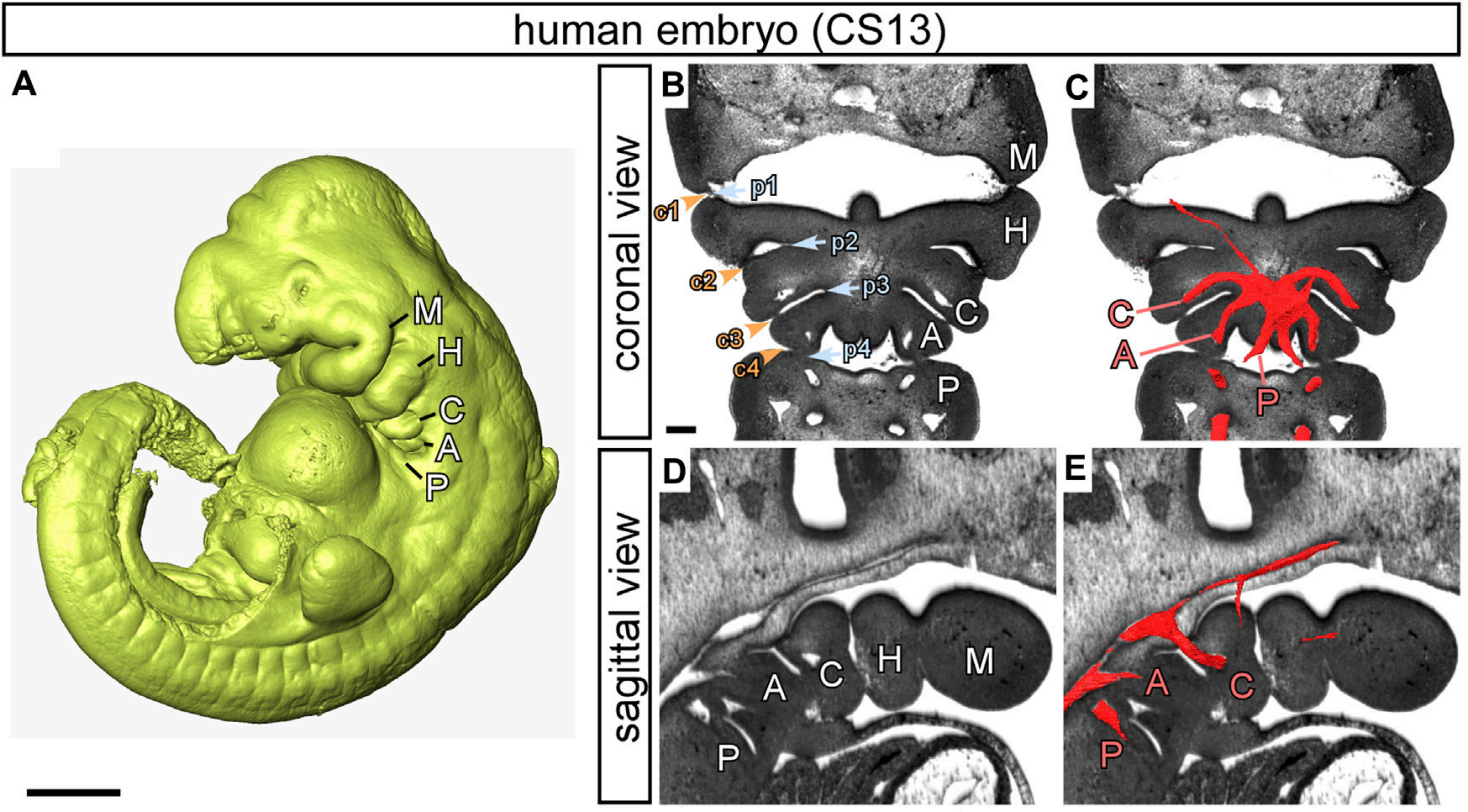
The pharyngeal arches in human embryos. Images prepared from high resolution episcopic microscopy datasets. **(A)** 3D reconstruction of a human embryo at CS13. The pharyngeal arches are visible: the mandibular (M), hyoid (H), carotid (C), aortic (A) and pulmonary (P). **(B–E)** High resolution episcopic microscopy images of a CS13 embryo in transverse **(B, C)** and sagittal **(D, E)** views. **(B, D)** The arches are labelled, and the endodermal pouches (p; blue text and arrowheads) and ectodermal clefts (c; orange text and arrowheads) are numbered **(B)**. **(C, E)** 3D reconstructions of the pharyngeal arch arteries have been overlaid on the images and labelled with the relevant letter in red to indicate the carotid (C), aortic (A) and pulmonary (P) arch arteries. Scale: 1 mm in A; 200 µm in B-E. Figure adapted from (Graham et al., 2023).

In the developing mammalian embryo, the pharyngeal arches emerge as a series of bulges, which develop in a cranial to caudal sequence along the lateral surface of the head (Graham and Smith, 2001) (Figure 1). These structures are made up of many cell types. They are lined by epithelia of endodermal origin on the inside, and of ectodermal origin on the outside. In between these linings, the arches are packed with neural crest cell-derived mesenchyme surrounding a core of mesoderm which is derived from the lateral plate mesoderm (Chapman et al., 1996). Each arch is separated from its neighbours by ectodermal clefts externally, and endodermal pouches internally, with the epithelial layers lining the clefts and pouches almost, but not quite, meeting (Shone and Graham, 2014). The pouches protrude to contact the overlying ectoderm, which invaginates to meet them, generating the pharyngeal clefts. It is the points of contact between the pouches and clefts that then define the limits of the arches. The first pouch separates the first and second arches. The second pouch then interposes between the second and third arches, and so on, with the eventual formation of four pouches. In mammals there are five pharyngeal arches. As the embryo develops, the arches are rapidly reorganised so that the segmented appearance disappears. The second arch expands caudally, while the more caudal arches concomitantly internalise (Poopalasundaram et al., 2023). Within the arches, the pharyngeal glands, such as the thymus, parathyroids, and ultimobranchial bodies, are developed from the pharyngeal endoderm. Interestingly, however, the epithelial reticulum of the thymus is of endodermal origin (Blackburn and Manley, 2004). The ectoderm gives rise to the epidermis, and the pharyngeal portion of the lateral plate mesoderm gives rise to skeletal muscles and to the endothelium of the pharyngeal arch arteries. The cells derived from the neural crest transform to provide skeletal tissues and the smooth musculature of the pharyngeal arch arteries (Graham et al., 2019).

The pharyngeal arches, and the arteries found within them, are often interchangeably referred to by name and/or number. For example, the pharyngeal arches may be referred to by name, such as the mandibular, hyoid, and carotid arches, while simultaneously the pharyngeal arch arteries are identified by number, usually 1 through 4 and 6, but are called simply ‘arches’ (Congdon, 1922; Romer, 1962; Kolesová et al., 2007). More recently, researchers have tended to only use numbers for both the pharyngeal arches and their arteries (Phillips et al., 2019; Hikspoors et al., 2022). As we will describe, this is confusing and can create problems, particularly for those dealing with congenital cardiac malformations. In our opinion, it is preferable to give names to the pharyngeal arches themselves, and for the forming arteries that exist within them, rather than numbers, so as to reflect the major structures to which they contribute. The first arch is therefore the mandibular arch, the second is the hyoid arch, and the third the carotid arch. The fourth is the aortic arch, with the fifth, or ultimate arch, being the pulmonary arch (Figure 1) (Graham et al., 2023). With this nomenclature, we are suggesting a terminology that works across the amniotes, in other words reptiles, birds, and mammals. The fourth aortic arch artery has previously been referred to as the systemic arch artery in amphibians (Romer, 1962) and the third, in reptiles, as the carotico-systemic arch artery (Goodrich, 1919). The systemic arteries, however, emerge at a later developmental stage, appearing subsequent to remodelling of the caudal arches. They form differently in reptiles, birds and mammals. It should also be considered that the caudal arches, apart from the ultimate pulmonary arch, all contribute to the systemic circulation. For all these reasons, we prefer the term aortic arch for the fourth pharyngeal arch and its artery.

## The arteries of the pharyngeal arches in human embryos.

**FIGURE 2:**
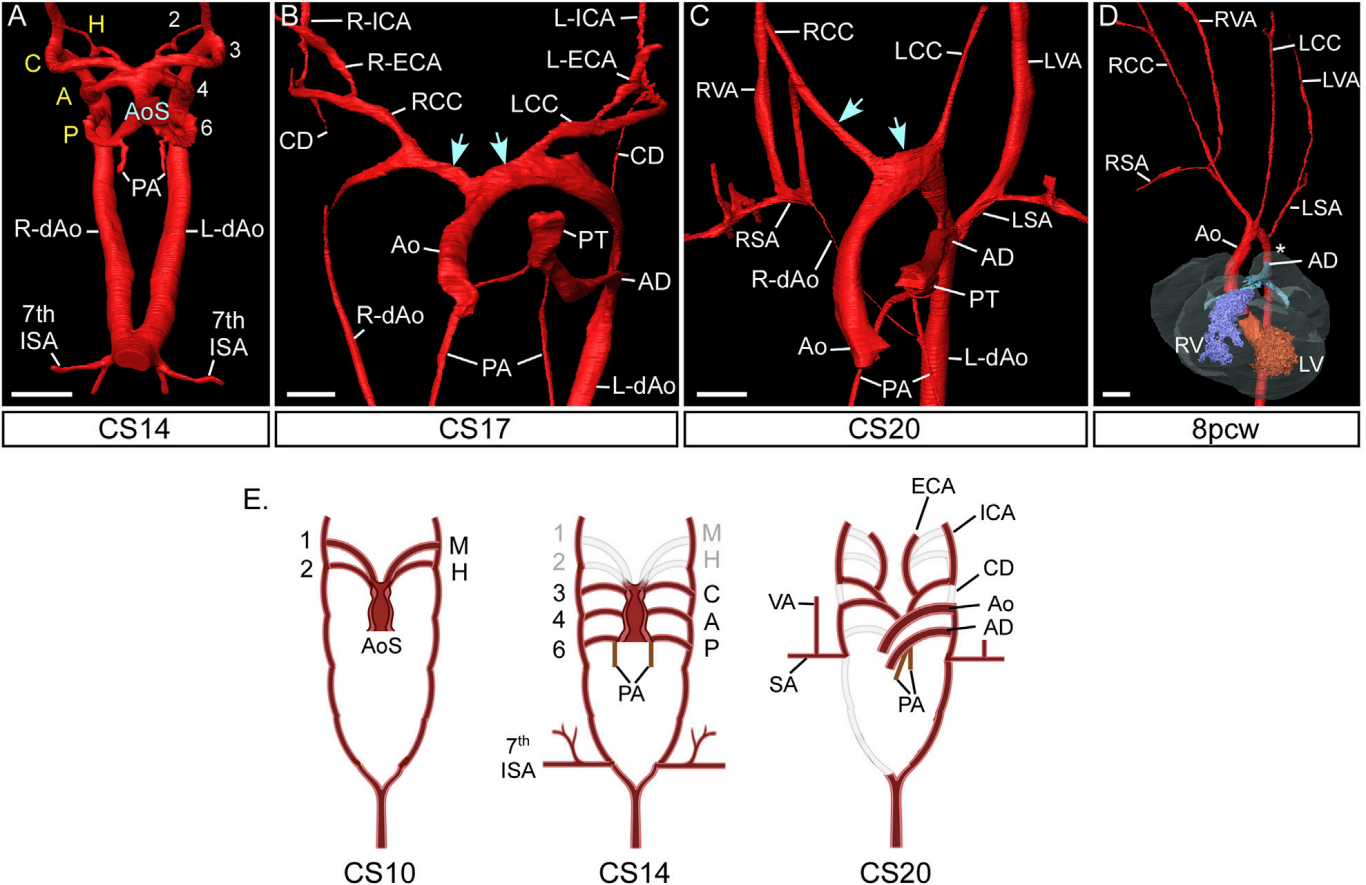
Morphogenesis of the pharyngeal arch arteries in human embryos. 3D reconstructions were made from high resolution episcopic microscopy **(A–C)** and micro-CT **(D)** datasets. **(A)** By the CS14 stage in human development, the caudal three pharyngeal arch arteries are symmetrical and of equal diameter. The arch arteries connect to the heart via the aortic sac. **(B)** By CS17 the aortic sac has been modified: the proximal part is now divided into the aorta and pulmonary trunk of the outflow tract, and the distal part forms right and left horns (arrows) that connect to the remodelling aortic arch arteries. The carotid ducts have involuted and the common, internal and external carotid arteries are apparent, and the right dorsal aorta is thinning. **(C)** Towards the end of the embryonic phase of development, CS20, the right subclavian artery is almost in its mature configuration with the right aortic arch artery joining to the right 7^th^ intersegmental artery. The remodelled horns of the aortic sac is shown (arrows). **(D)** Arch artery morphogenesis is complete by the 8pcw fetal stage. The region of the aorta between the left subclavian artery and arterial duct, the isthmus, is indicated (asterisk). **(E)** Schematic representation of pharyngeal arch artery remodelling in the human, with the key stages shown at CS10, CS14 and CS20. At CS10 only the first mandibular and second hyoid arch artery have formed, and these have remodelled by CS14. Abbreviations: A, aortic PAA; AD, arterial duct; Ao, aorta; AoS, aortic sac; C, carotid PAA; CC, common carotid artery; CD, carotid duct; CS, Carnegie Stage; dAo, dorsal aorta; ECA, external carotid artery; H, hyoid PAA; ICA, internal carotid artery; L, left; LSA, left subclavian artery; P, pulmonary PAA; PA, pulmonary arteries; pcw, post conception weeks; PT, pulmonary trunk; R, right; SA, subclavian artery; V, ventricle; VA, vertebral artery. Scale: 200 µm in AB; 500 µm in C; 1 mm in **(D)**. Figure adapted from (Anderson and Bamforth, 2022). Panel E created with BioRender.com.

During the sequence of normal development, an arch artery forms within each of the pharyngeal arches connecting the aortic sac to the paired dorsal aortas. As they form, the arteries develop symmetrically and sequentially in a cranial to caudal manner. They then rapidly remodel so that, by the fetal stage, they have transformed into the asymmetric arrangement as seen postnatally (Figure 2). In the human, the heart is identifiable at Carnegie Stage (CS) 9 (Hikspoors et al., 2022), when the embryo is around 26 days after conception (Table 1) (O'Rahilly and Muller, 2010). By CS10, the first set of arteries form in the mandibular arch, exit the primary heart tube cranially, and loop posteriorly to join the paired dorsal aortas. These vessels have been described differently in the mouse as the paired ventral aortas (Hiruma et al., 2002) but this is not equivalent in humans (Congdon, 1922). The second, or hyoid, arteries form shortly afterwards at CS11. These two sets of arteries quickly remodel such that, by CS13, they have been interrupted. The distal parts then form the mandibular and hyoid arteries, with the proximal parts contributing to the external carotid arteries (Hiruma et al., 2002; Anderson and Bamforth, 2022).

**TABLE 1:** Carnegie stages and comparable mouse development stages post fertilisation. Adapted from (Kaufmann, 1992; O'Rahilly and Muller, 2010).

Carnegie stage	Days post fertilisation	Weeks post fertilisation	Equivalent mouse stage
10	28–30	4	E8.5 - E9.0
11	28–30		E9.0 - E9.5
12	29–31		E9.5 - E10.25
13	30–33		E10.25 - E10.5
14	33–35	5	E10.5
15	35–37		E11.0
16	37–40		E11.5
17	39–42	6	E12.0
18	42–45		E12.3 - E13.5
19	45–47		
20	47–50	7	E13.5 - E14.0
21	49–52		
22	52–55		
23	53–58	8	

(E, embryo stage).

The arteries of the third, or carotid, arch can already be recognised at CS12, with the arteries of the fourth aortic arch also evident at this stage. Both the carotid and aortic arch arteries are well formed by CS13 (Hikspoors et al., 2022). The ultimate pair of arteries are formed within the pulmonary arch between CS14 and CS15 (Figure 2A) and this morphological event is variable between embryos as some developmental steps take two Carnegie stages to be completed (McBride et al., 1981; Rana et al., 2014; Anderson and Bamforth, 2022; Hikspoors et al., 2022). By this stage of development, the regression of the cranial arteries means that three pairs of pharyngeal arch arteries are present (Anderson and Bamforth, 2022; Hikspoors et al., 2022). They have retained their symmetry, and are of approximately equal diameter, encircling the trachea-oesophageal pedicle to connect the aortic sac with the paired dorsal aortas. These caudal three sets of arteries then themselves undergo a dramatic and rapid process of remodeling. This coincides with the onset of septation and rotation of the outflow tract. It involves thinning and eventual disappearance of the right pulmonary pharyngeal arch artery, and similar attenuation of the right dorsal aorta. By CS15, the aortic sac has itself remodelled to form cranial horns that connect the ascending aorta to the aortic and carotid pharyngeal arch arteries. By CS17, the artery of the left pulmonary arch has expanded to form the arterial duct, which functions as a shunt to divert right ventricular blood away from the non-functioning lungs of the fetus (Figures 2B,C). The right and left pulmonary arteries, which take deoxygenated blood to the developing lungs, are now forming within the pharyngeal mesenchyme, but take their origin from the proximal portions of the pulmonary arch arteries, which themselves take their origin from the caudal part of the aortic sac.

During these stages, the arteries of the aortic arches have remodelled in distinct fashion on the right and left sides to achieve their mature configuration (Figure 2D). On the right side, the artery of the aortic arch becomes part of the right subclavian artery, joining with the right seventh cervical intersegmental artery. The developing artery takes its origin from the right cranial horn of the aortic sac, which becomes the brachiocephalic trunk. On the left side, in contrast, the artery of the aortic arch forms the part of the definitive transverse aortic arch between the origins of the left common carotid and left subclavian arteries. The proximal part of the arch, between the brachiocephalic trunk and the left common carotid artery, is formed from the left cranial horn of the aortic sac.

The developing subclavian arteries incorporate the seventh cervical intersegmental arteries on both sides. These intersegmental vessels originally take their origin close to the point where the paired dorsal aortas had initially united to form a single vessel (Figures 2A,E). During the process of remodelling, as the embryo grows, the right dorsal aorta regresses caudal to the origin of the seventh intersegmental artery. Concomitant with this growth, the heart itself descends relative to the location of this fixed segmental vessel (Anderson and Bamforth, 2022). On the right side, the cervical intersegmental artery then incorporates part of the right dorsal aorta as it joins the artery of the right embryonic aortic arch to form the right subclavian artery (Figures 2C,E). The left subclavian artery is formed exclusively from the left seventh cervical intersegmental artery. With the growth of the embryo, this artery migrates cranially along the left dorsal aorta, crossing the origin of the arterial duct by the so-called “castling” movement. By these means it achieves its final position on the definitive aortic arch distal to the origin of the left common carotid artery (Figure 2D). The component of the dorsal aorta between the origin of the subclavian artery and the arterial duct is then known as the aortic isthmus.

The arteries of the carotid arches remodel on each side into the common and internal carotid arteries. The common carotid arteries are formed as the proximal parts of the arteries of the carotid arch elongate as the embryo grows along the cranio-caudal axis (Anderson and Bamforth, 2022). With these changes, the segments of the dorsal aortas originally positioned between the dorsal attachments of the arteries of the carotid and aortic arches, the carotid ducts, involute (Figures 2B,E). The internal carotid arteries are then formed from the distal components of the arteries of the carotid arches, along with the cranial parts of the dorsal aortas. The external carotid arteries are initially formed from the proximal parts of the arteries of the hyoid arches. These vessels also elongate as the embryo grows rapidly between CS13 and 20 (Hiruma et al., 2002; Anderson and Bamforth, 2022).

## Evolution of the arteries of the pharyngeal arches

**FIGURE 3:**
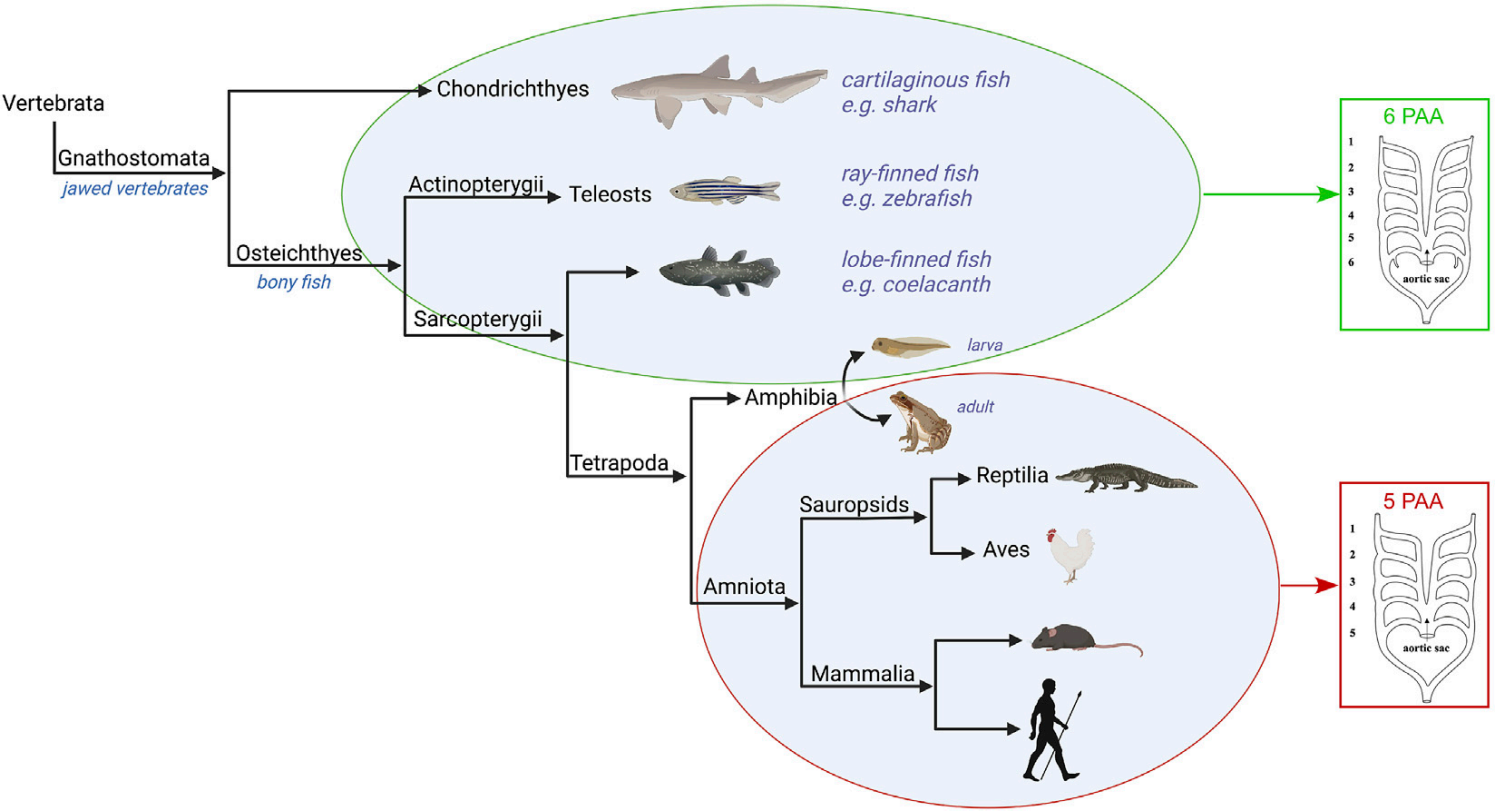
Evolution of the pharyngeal arch arteries in the jawed vertebrates. The Gnathostomes all develop six paired pharyngeal arch arteries apart from the amniotes which develop five. The amphibia are examples of tetrapods that have six pharyngeal arch arteries in their larval water-based form, but only five pairs in the adult land-based form. Figure created with Biorender.com.

The basic pattern of the pharyngeal arch arteries in jawed vertebrates, or gnathostomes, is typically understood to include six pairs (Figure 3). This number of arteries does not match the number of pharyngeal arches, since the ancestral condition for the gnathostomes is to have seven pharyngeal arches. Instead, the number of arch arteries matches the number of pharyngeal pouches, of which there were six. Indeed, the correlation between the number of pouches and arch arteries also extends to the jawless vertebrates. So, in lampreys there are nine arches, but eight pouches and eight sets of arteries. It has also recently been shown, in zebrafish, that the formation of the pharyngeal pouches coincides with the emergence of the arteries contained within the arches (Mao et al., 2021). The pouches express bone morphogenetic proteins (BMPs). It is these signalling molecules that are the primary cues for promoting the initial development of the arteries within the pharyngeal mesoderm. The alignment of formation of the arteries with the pouches then establishes a correspondence with the internal gills, which are also derived from the pouches (Warga and Nüsslein-Volhard, 1999; Gillis and Tidswell, 2017). In different clades, as development proceeds, we see loss of some of the cranial pharyngeal arteries. In many jawed vertebrates, the first arch artery recedes, while in species such as zebrafish, it is the second that recedes. In all the species, nonetheless, the four most caudal of the arteries always develop in robust fashion. It is these arteries that are associated with the gills.

Significant changes in the organisation of the arches and their arteries are found with the evolution of the tetrapods (Figure 3). This is associated with the transition from respiration via gills to air-breathing using lungs. While lungs are believed to have evolved prior to the emergence of the tetrapods, it is within this group that paired lungs assume a dominant role in the respiratory system (Cupello et al., 2022). These changes reflect the transition of vertebrates from an aquatic life to terrestrial living, and can be observed in the metamorphosis of contemporary amphibians with bi-phasic ontogeny. Their water-dwelling larval forms take oxygen from the water, whereas terrestrial adults use lungs for gas exchanges. The metamorphosis involves not only the transformation of the gill-supporting skeleton, but also changes in the associated pharyngeal arches and their contained arteries. This is most clearly seen in the Anurans, one of the main orders of the amphibia. Anurans develop six pairs of pharyngeal arch arteries, of which the first two cranial pairs, the mandibular and hyoid, disappear very early in development (Millard and Adamson, 1943; Nieuwkoop et al., 1994; Kolesová et al., 2007). This, therefore, leaves four pairs of pharyngeal arch arteries up to the pre-metamorphic stage. During metamorphosis, the pharyngeal arch arteries remodel to form the mature arch arteries of the adult. The exterior gills disappear, the carotid ducts involute to form the internal carotid arteries, the fourth arch arteries form the aortic arch, while the fifth arch arteries disappear, and the pulmonary arch artery carries deoxygenated blood to the skin and newly developed lungs (Millard and Adamson, 1943; Kolesová et al., 2007). The development of the pharyngeal arches and the associated arteries in amphibians, however, is distinct from other tetrapods and the amniotes such that direct comparisons are not possible. Published articles frequently focus on blood vessels that are not equivalent to the arteries which form within the pharyngeal arches of amniotes. For example, stages 50–66 examined in frogs (Jourdain, 1894; Grobbelaar, 1924; Millard and Adamson, 1943; Kolesová et al., 2007) are much later than the stages encompassing formation of the pharyngeal arches in amniotes, which would be equivalent to stages 22–35 (Fisher et al., 2023). In *Xenopus*, the first two pouches from at stage 22, the third pouch at stage 27, the fourth pouch at stage 28 and the last pouch at stage 35. The differences have been attributed to the formation of external gills, the anterior pharyngeal arch skeleton being adapted to feeding, and the posterior arch skeleton being fused to form a branchial basket (Pugener et al., 2003; Square et al., 2015).

A key feature of the tetrapods is the robust development of the pulmonary arteries, which carry deoxygenated blood to the skin and newly developed lungs (Dzialowski, 2018; Cupello et al., 2022). These are the ultimate pair of pharyngeal arch arteries. A notable feature of these vessels is that they develop caudal of the last pharyngeal pouch. They are in a distinct position relative to the most caudal artery of non-tetrapods, which lie cranial of the last pouch. It follows, therefore, that the most caudal pharyngeal artery of tetrapods is not homologous with the most caudal artery of other vertebrates. In mammalian embryos the pulmonary arch arteries are the first to remodel, with the artery on the right disappearing, and the artery on the left forming the arterial duct. This allows for the right-to-left shunting of blood from the right ventricle to the systemic circulation, thereby during gestation bypassing the non-functioning lungs. After birth, with the onset of air breathing, the arterial duct closes within 48 h. The deoxygenated blood is then fed to the lungs for oxygenation, and is returned to the left ventricle for pumping to the rest of the body. This is not always the case, however, in many non-mammalian amniotes in which both arterial ducts remain patent, at least, up to birth or hatching, for example, in the chick embryo (White, 1974).

The evolution of the amniotes resulted in their embryos being freed from development in an aquatic environment. This is reflected in still further significant changes in the pharyngeal arches and their arteries. Thus, the number of arches is reduced to five, with formation of only four pharyngeal pouches. As was the case with the other tetrapod clades, the number of arteries formed exceeds by one the number of pharyngeal pouches. The finding of four pouches, with five sets of arteries, is then conserved throughout the amniotes (Hiruma and Hirakow, 1995; Hiruma et al., 2002). In the four cranial arches, the artery forms cranial to its associated pouch, again as found in all other vertebrates. In the last arch, however, which is the pulmonary arch, the arteries are formed caudal to the last pharyngeal pouch. If a pharyngeal arch would be morphologically defined by the presence of a cranial as well as caudal border formed by ectodermal clefts (externally) and endodermal pouches (internally), we then could call into question the classification of the pulmonary arch arteries as true pharyngeal arch arteries and their homology with the most caudal arteries seen in other gnathostomes.

Although all land-based amniotes have five pairs of pharyngeal arch arteries, the morphogenesis is markedly different in the various clades. As described above, in mammals the carotid arteries arise from the brachiocephalic artery on the right, and directly from the aortic arch on the left. It is the artery of the left embryonic aortic arch that forms part of the definitive transverse aortic arch, which then joins to the left-sided dorsal aorta (Figure 2). In birds, the symmetrical appearance of the arch arteries in a cranial to caudal sequence is much the same as in mammals (Hiruma and Hirakow, 1995), with the two cranial sets becoming interrupted by the time the final two caudal sets have formed. It is the artery of the embryonic left aortic arch, however, that regresses at the same stage as the carotid ducts in birds. The artery of the embryonic right aortic arch then expands to form a definitive right-sided aortic arch, connecting to the right dorsal aorta. The ventral aorta then gives origin to paired brachiocephalic trunks, each dividing into subclavian and carotid arteries (Mackay and Cleland, 1887).

In most reptiles, as seen in the Lacertidae, the heart is univentricular, from which two aortas and a pulmonary trunk emerge. In the embryo the paired arterial ducts drain into the paired dorsal aortas. In the adult, the carotid arch arteries branch from the systemic aorta, and the carotid ducts persist (Zug, 1971). Crocodiles, however, have two ventricles similar to birds and mammals. Whereas in birds the right aortic arch artery persists, and it is the left in mammals, in crocodiles there are two aortas directing blood from the heart to distinct regions of the body (Poelmann et al., 2017). The right-sided aortic arch arises from the left ventricle and takes oxygenated blood from the lungs to the head and body wall, while the left-sided aorta arises from the right ventricle and delivers blood to the organs (Cook et al., 2017). In the crocodilian embryo, the pulmonary trunk supplies deoxygenated blood to the paired dorsal aorta via both left and right arterial ducts (Dzialowski, 2018).

### Congenital cardiovascular malformations

The mammalian pharyngeal arch arteries, during their development, take origin from the aortic sac, which arises at the margins of the pericardial cavity from the outflow tract of the heart. This structure is the arterial pole of the primary heart tube. With ongoing development, the addition of non-myocardial tissues to the outflow tract produces the intrapericardial arterial trunks, which then feed separately the cranial and caudal components of the initial aortic sac. If development proceeds normally, deoxygenated blood is then pumped from the right ventricle through the pulmonary trunk to the lungs, via the pulmonary arteries, for oxygenation. Oxygenated blood is returned to the left side of the heart and is pumped to the systemic circulation via the aorta and the transverse aortic arch.

**FIGURE 4:**
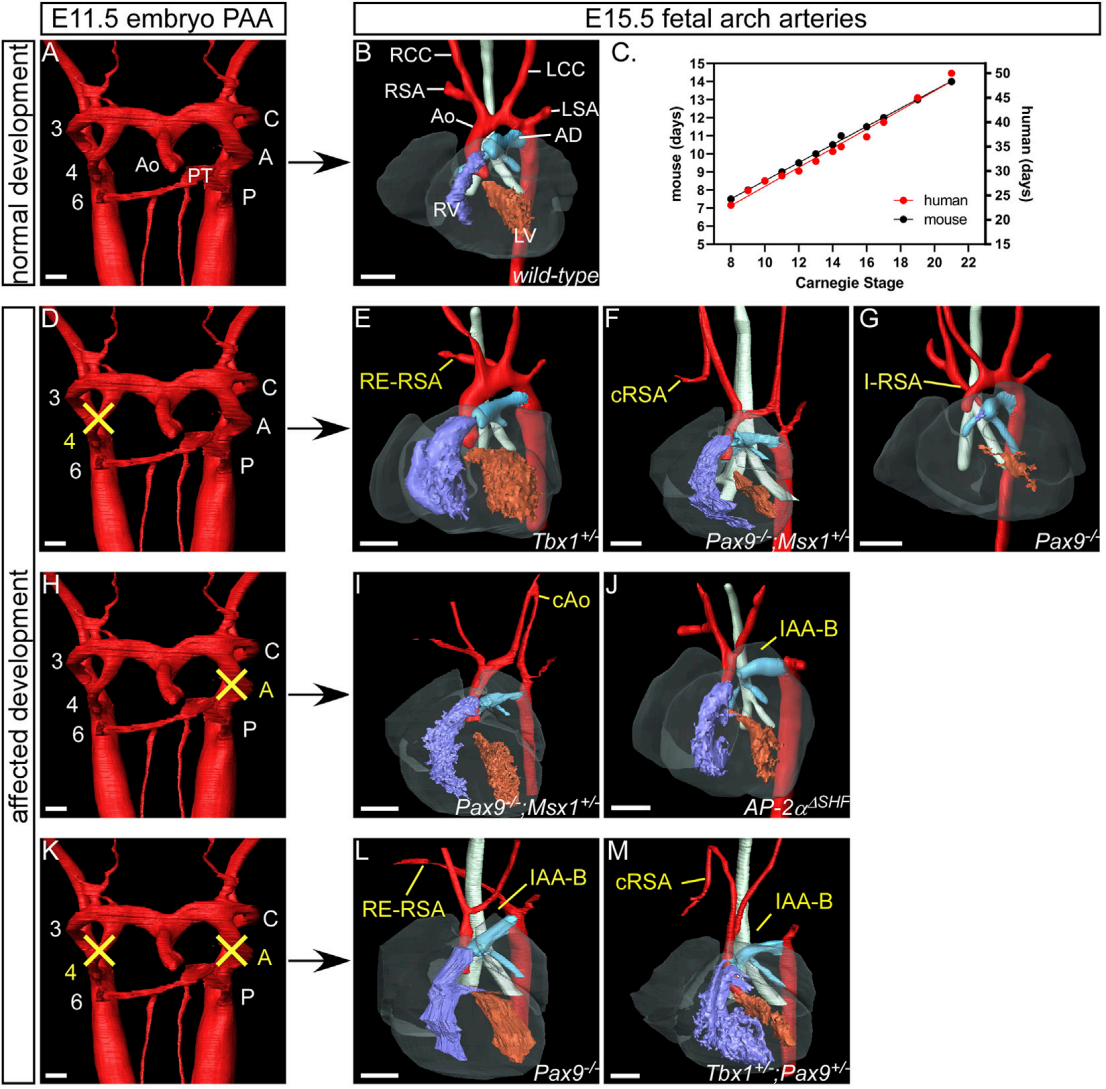
Examples of human congenital cardiovascular malformations affecting the aortic arch arteries seen in mutant mouse models. 3D reconstructions were made from high resolution episcopic microscopy **(A,D,H and K)**, MRI **(B,E,G and J)** and micro-CT **(F,I,L,M)** datasets. **(A)** The pharyngeal arch arteries are beginning to remodel in the normal mouse embryo at E11.5 with septation of the outflow tract into the aorta and pulmonary trunk. **(B)** By the fetal stage (E15.5) the aortic arch arteries are in their asymmetric mature configuration. The right and left aortic (fourth) pharyngeal arch arteries contribute to the right subclavian artery and the transverse aortic arch respectively. **(C)** Graph to illustrate the comparison between mouse (Theiler, 2013) and human (O'Rahilly and Muller, 2010) developmental days with Carnegie Stages. **(E-G), (I, J), (L, M)** Gene mutations in mouse models are indicated in the panels. **(D)** Failure of the right aortic pharyngeal arch artery can result in retro-esophageal right subclavian artery (RE-RSA; **(E)**, cervical origin of the RSA (cRSA; **(F)** or isolated RSA (I-RSA; **(G)**. **(E)** If the left aortic pharyngeal arch artery does not form, this can result in cervical origin of the aorta (cAo; **(I)** or an interrupted aortic arch type B (IAA-B; **(J)**. **(K)** Bilateral failure of the aortic pharyngeal arch arteries results in RE-RSA and IAA-B **(L)** or cRSA and IAA-B **(M)** occurring simultaneously. Abbreviations: A, aortic pharyngeal arch artery; AD, arterial duct; Ao, aorta; AoS, aortic sac; C, carotid pharyngeal arch artery; CC, common carotid artery; CD, carotid duct; dAo, dorsal aorta; ECA, external carotid artery; H, hyoid pharyngeal arch artery; ICA, internal carotid artery; L, left; LSA, left subclavian artery; P, pulmonary pharyngeal arch artery; PAA, pharyngeal arch arteries; R, right; SA, subclavian artery; V, ventricle, ΔSHF, deleted from the second heart field. Scale: 100 µm in A,C,G,J; 500 µm in B,D-F,H,I,K. Figure adapted from (Phillips et al., 2019; Johnson et al., 2020; Stothard et al., 2020; Khasawneh et al., 2021).

**FIGURE 5:**
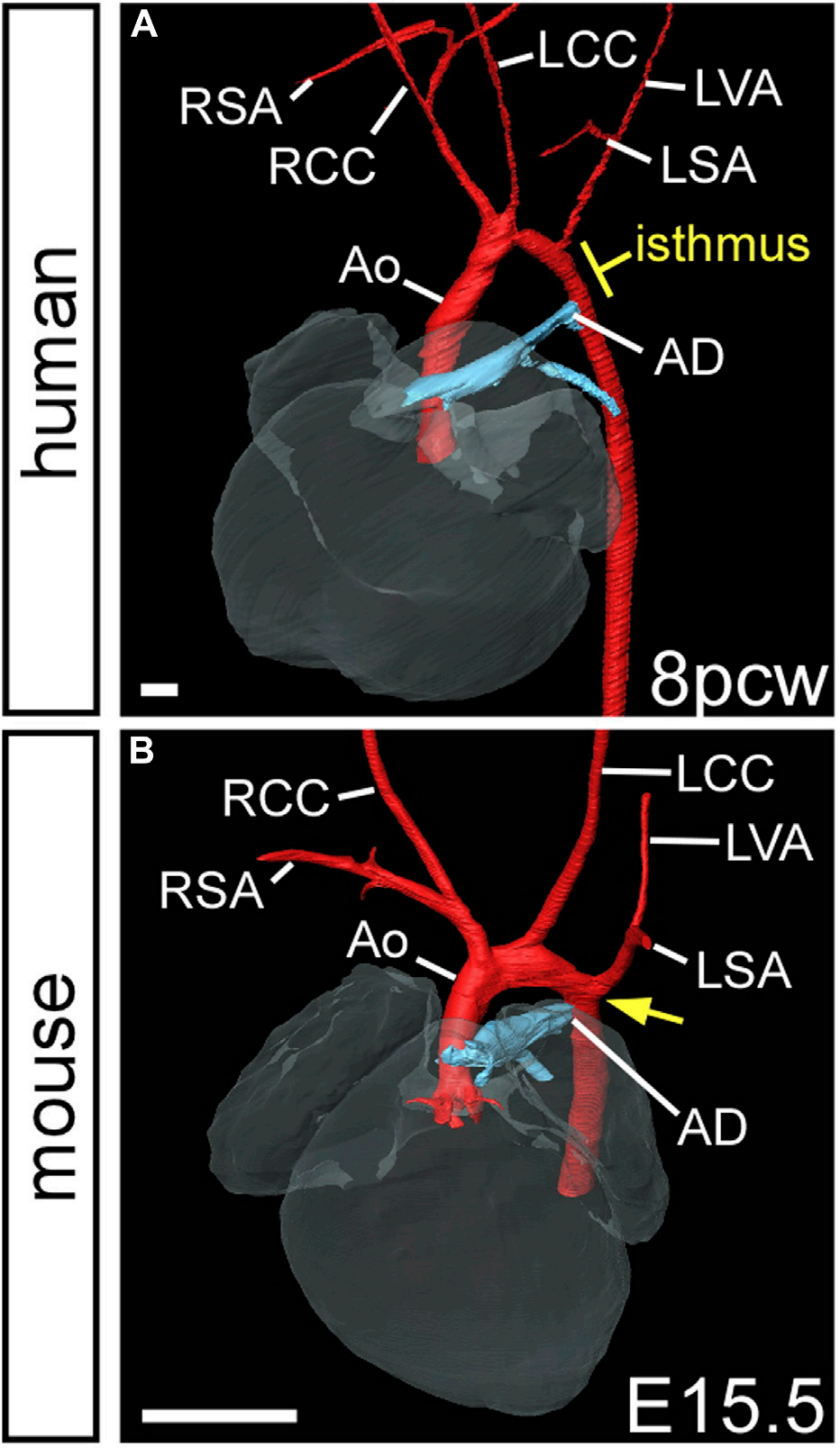
Position of the left subclavian artery in humans and mice. 3D reconstructions were made from micro-CT datasets. **(A)** In the human fetus, the left subclavian artery is located in a relatively higher position on the aorta than the arterial duct. The intervening segment is known as the isthmus. **(B)** In the mouse fetus, the arterial duct inserts into the aorta opposite the left subclavian artery (yellow arrow). Abbreviations: Ao, aorta; LCC, left common carotid artery; LSA, left subclavian artery; LVA, left vertebral artery; pcw, post conception weeks; RCC, left common carotid artery; RSA, left subclavian artery. Scale, 500 µm. Figure adapted from (Anderson and Bamforth, 2022).

If the bilaterally symmetrical pharyngeal arch arteries do not form or remodel correctly, congenital cardiovascular malformations occur. Here we show developmental defects of the arch arteries in genetically altered mouse models using high resolution imaging techniques (Figure 4). The mouse is a good model to study human arch artery development as general development, and the processes in pharyngeal arch artery formation and remodelling, are directly comparable (Anderson and Bamforth, 2022; Hikspoors et al., 2023) (Figure 4C; Table 1). We, and others, have shown that mice with mutations in transcription factors such as *Tbx1* (Jerome and Papaioannou, 2001; Anderson and Bamforth, 2022), *Pax9* (Phillips et al., 2019; Anderson and Bamforth, 2022), *Gbx2* (Calmont et al., 2009; Stothard et al., 2020), *Msx1* (Khasawneh et al., 2021) and *AP-2α* (Johnson et al., 2020) develop severe defects in arch artery development that are also seen in humans. *TBX1* is one of the genes deleted in 22q11 deletion syndrome but is likely to be the most important mutated gene causing the observed arch artery defects in patients (Gong et al., 2001), and a patient hemizygous for *PAX9* was identified with interruption of the aortic arch (Santen et al., 2012). Retro-esophageal origin of the right subclavian artery is found when the right aortic arch artery fails to form (Figure 4D) and there is regression of the component of the right dorsal aorta cranial to the origin of the right seventh intersegmental artery. The persisting caudal component of the right dorsal aorta then provides the origin of the right subclavian artery from the left-sided descending aorta, with the right-sided subclavian artery taking its own origin distal to that of the left subclavian artery (Figure 4E). The right subclavian artery must then cross the midline in retroesophageal fashion to reach the right arm. This lesion is relatively common in the general population, with an incidence of 1%, although the incidence increases to almost 25% in Downs syndrome (Scala et al., 2015). The lesion does not always cause symptoms, although it can produce dysphagia. Cervical origin of the right subclavian artery is found when there is persistence of the right carotid duct in the absence of the right aortic arch artery (Figure 4F). The artery of the right carotid arch then carries blood via the persisting carotid duct to the right subclavian artery, which continues to be formed by the right seventh intersegmental artery (Ezzeldin et al., 2018). The right subclavian artery can also be isolated when, in the absence of the right aortic pharyngeal arch artery, the artery of the right pulmonary arch provides the connection with the right intersegmental artery (Figure 4G) (Nath et al., 1981; McElhinney et al., 1998). This is problematic, as the isolated subclavian artery will carry deoxygenated blood from the right ventricle to the right arm. Similar lesions can involve the left subclavian artery when the definitive aortic arch itself is right-sided. If the artery of the embryonic left aortic arch fails to form, or disappears during the phase of remodeling (Figure 4H), two distinct defects can occur. If the carotid arch artery persists in the absence of the left aortic arch artery, along with persistence of the left carotid duct, an aorta with cervical origin is formed (Figure 4I). Alternatively, the definitive aortic arch becomes interrupted between the origins of the left common carotid and left subclavian arteries. This is termed a type B interruption (Figure 4J). This defect is very rare in the general population, but when found, half of all cases are identified to be in the setting of the 22q11 deletion syndrome (Van Mierop and Kutsche, 1986; Lewin et al., 1997; Boudjemline et al., 2001). Interruption of the aortic arch type B can exist with retro-esophageal or cervical origin of the right subclavian artery when both left and right aortic pharyngeal arch arteries fail to form (Figures 4K–M) (Kutsche and Van Mierop, 1984). Not all cases of aortic arch interruption, however, are of this B type. The arch may be interrupted at the isthmus. This type of interruption, known as type A, is part of a spectrum that includes aortic coarctation. In this regard, it is of note that the developmental remodeling of the left subclavian artery in the human is different than seen in the mouse. In the human, the left subclavian artery, as already explained, migrates cranially along the dorsal aorta, passing the junction of the arterial duct in the castling movement to produce the segment of the aorta known as the isthmus (Figure 5A). In mice, however, the left subclavian artery lies directly opposite the site of union of the dorsal aorta and the arterial duct (Figure 5B). This implies less “castling” than is seen during human development. Coarctation at the isthmus forms a shelf of ductal tissue, with the narrowing restricting the flow of blood from the heart to the lower body. It is very rare to find interruption of the aortic arch between the origins of the carotid arteries, known as type C. The finding of this variant implies interruption between the two initial cranial horns of the aortic sac. Irrespective of their site, all the types of interruption can be further complicated by anomalous retro-esophageal origin of the subclavian arteries, and by interruption in a right-sided rather than a left-sided aortic arch.

**FIGURE 6:**
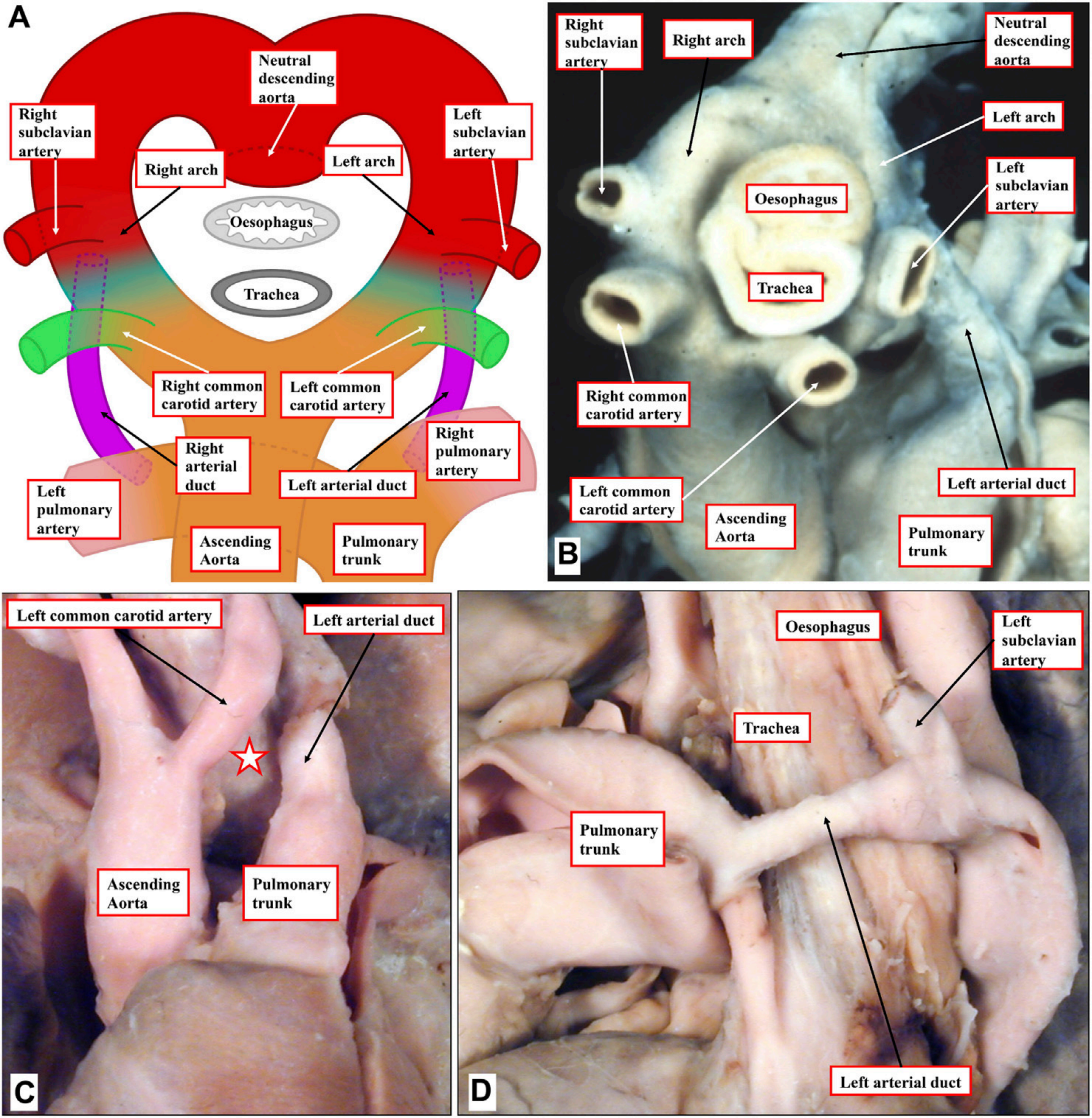
Human congenital cardiovascular malformations: aortic rings. **(A)** The hypothetical double aortic arch model as proposed by Edwards. **(B)** An example from a human patient displaying a close cousin of the hypothetical model, lacking only a right-sided arterial duct. **(C, D)** A human heart with an incomplete double arch. **(C)** Absence of the anterior left-sided arch (white star with red borders). **(D)** Lateral view shows the left-sided duct and the retroesophageal component of the left arch, which encircles the trachea-esophageal pedicle.

It is also possible for the bilaterally symmetrical arrangement of the arch arteries to persist, rather than involute. Persistence of the primordia of the arches on both sides produces the vascular rings, which when severe can produce significant esophageal constriction. All are well explained on the basis of the so-called hypothetical double arch (Figure 6A) (Edwards, 1948). This model shows that both arteries of the embryonic aortic arches persist, giving rise on each side cranially to common carotid and subclavian arteries, but it is the abnormal persistence of the distal segment of the embryonic right dorsal aorta that would lead, in an otherwise normal situation, to a double aortic arch. Caudally, each arch gives rise to an arterial duct, representing the proximal parts of the arteries of the pulmonary arches. To the best of our knowledge, the hypothetical perfect double arch has yet to be described, but multiple examples exist with the bilateral arches supplying both brachiocephalic arteries, and with one arch giving rise caudally to an arterial duct (Figure 6B). Parts of the double arch system, however, can partially involute and persist as fibrous strands. These variants can still produce symptomatic esophageal constriction (Figures 6C,D). It can be difficult in the clinical setting to identify the fibrous components in the constricting rings.

**FIGURE 7:**
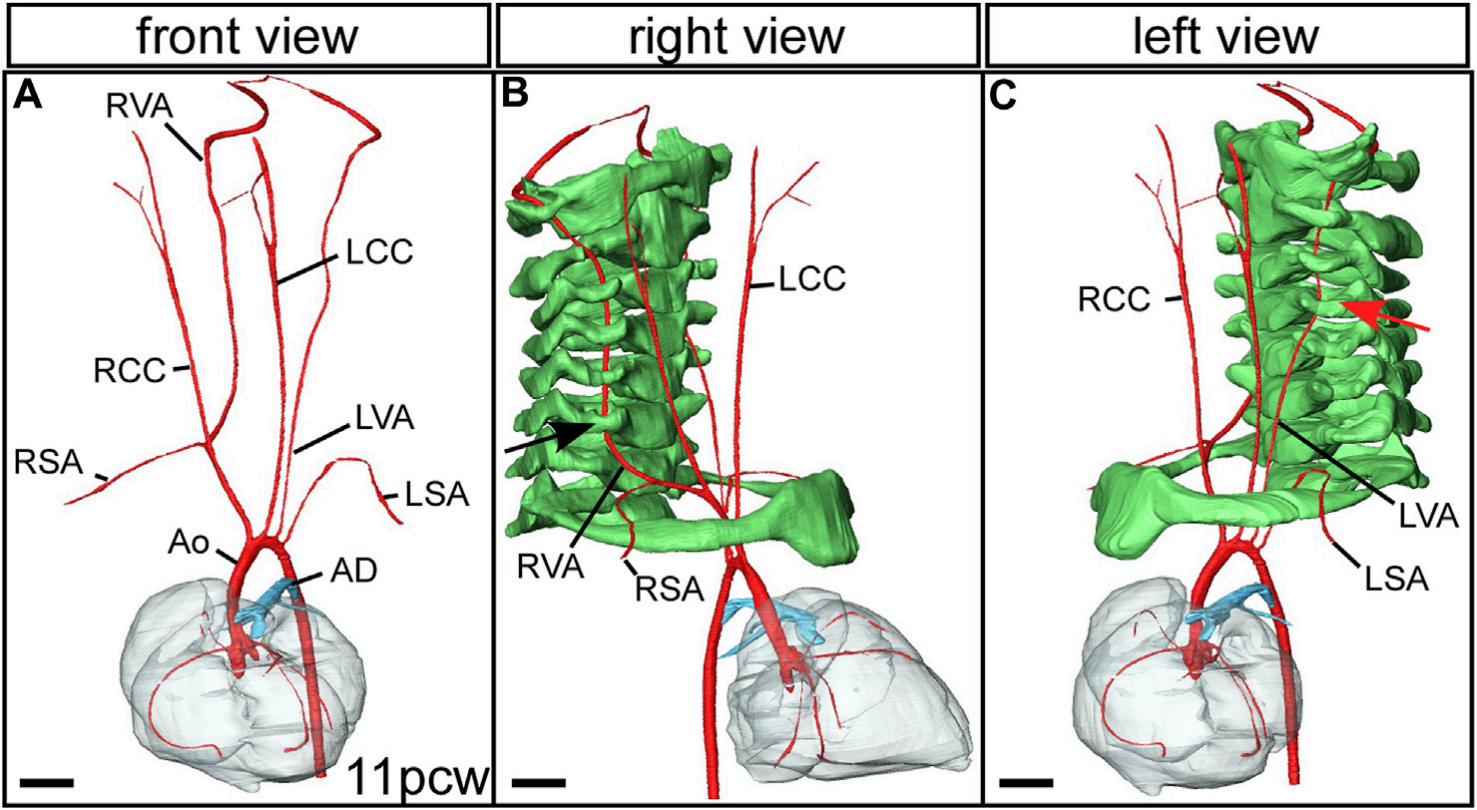
Aberrant vertebral artery morphogenesis. 3D reconstructions were made from micro-CT datasets of an 11pcw human fetus. **(A)** The arch arteries are shown, with the left vertebral artery arising aberrantly from the transverse aortic arch instead of from the left subclavian artery (compare with Figure 2D). The right vertebral artery comes off the right subclavian artery as usual. Right **(B)** and left **(C)** views with the cervical vertebrae and first rib included (coloured green). The right vertebral artery enters the foramen of the sixth cervical vertebra (black arrow). **(C)** The left vertebral artery enters the foramen of the fourth cervical vertebra (red arrow). Abbreviations: AD, arterial duct; Ao, aorta; LCC, left common carotid artery; LSA, left subclavian artery; LVA left vertebral artery; pcw, post conception weeks; RCC, right common carotid artery; RSA, right subclavian artery; RVA, right vertebral artery. Scale, 1 mm. Figure adapted from (Anderson and Bamforth, 2022).

Additional relatively common and mostly asymptomatic lesions can also result from variations in remodelling of the arch arteries. The vertebral arteries, which take blood to the head, usually run cranially from the subclavian arteries (Figures 2C,D; Figure 7A), entering the transverse foramen of the sixth cervical vertebrae (Figure 7B). An example of aberrant positioning of the left vertebral artery is found when the artery arises directly from the aortic arch between the left common carotid and left subclavian arteries. The artery then runs cranially, entering the vertebral column through the foramen of the fourth cervical vertebra (Figure 7C) (Anderson and Bamforth, 2022).

### The “fifth” arch artery in mammals

For many years now, paediatric cardiologists have diagnosed malformations of the extrapericardial arterial pathways on the presumption that there were initially six sets of pharyngeal arch arteries. This concept was based on the premise that the fifth set of arteries was vestigial, or formed only transiently before regressing. There is no evidence of which we are aware to substantiate the notion of formation of the fifth pair of pharyngeal arch arteries in mammals. This concept of six pairs of pharyngeal arch arteries in humans would demand the formation of an additional set of accompanying pharyngeal arches with pouches, and again, there is no developmental evidence of this feature.

**FIGURE 8:**
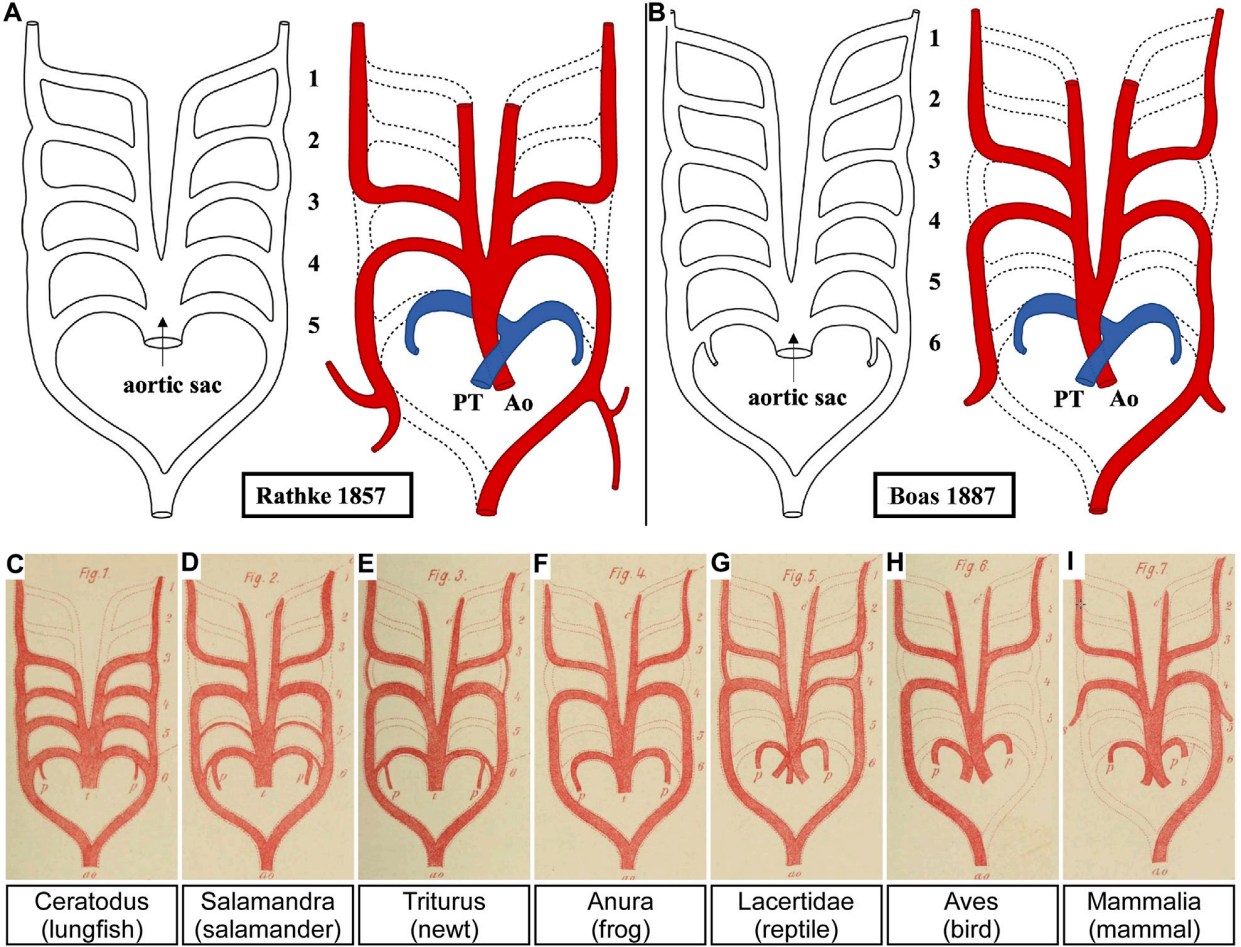
The five *versus* the six pharyngeal arch artery model. **(A)** The original pharyngeal arch artery plan for humans contained only five pairs of pharyngeal arch arteries. Here we have adapted Rathke’s original drawing to re-position the aorta and pulmonary trunk of the outflow tract. **(B–I)** Boas proposed that all jawed vertebrates, including humans **(B)**, must initially have six pharyngeal arch arteries. This view has prevailed despite no evidence to confirm this. Adapted from (Rathke, 1857; Boas, 1887).

The original account of the pharyngeal arches and their arteries showed human embryos with only four pouches and five sets of pharyngeal arch arteries (His, 1885), but a hypothetical schematic drawing of the remodelling mammalian pharyngeal arch arteries was shown by Rathke in 1857 (Figure 8A) (Rathke, 1857). This view, which matches perfectly with our own current findings, seems to have been challenged on the basis of a study in comparative anatomy comparing lungfish, amphibians, reptiles, birds and mammals (Figures 8B–I) (Boas, 1887). Boas postulated that all vertebrates developed four persisting pharyngeal arch arteries. He opined that, if the pulmonary arches were the ‘fifth’ in amniotes, their body plan had changed. For that reason, he claimed that a fifth set should temporarily have existed also in amniotes. Although this theory of a six-arch model was not met with agreement from all anatomists of the time (Lewis, 1906; Reagan, 1912; Congdon, 1922), the evolutionary line of thought prevailed.

**FIGURE 9:**
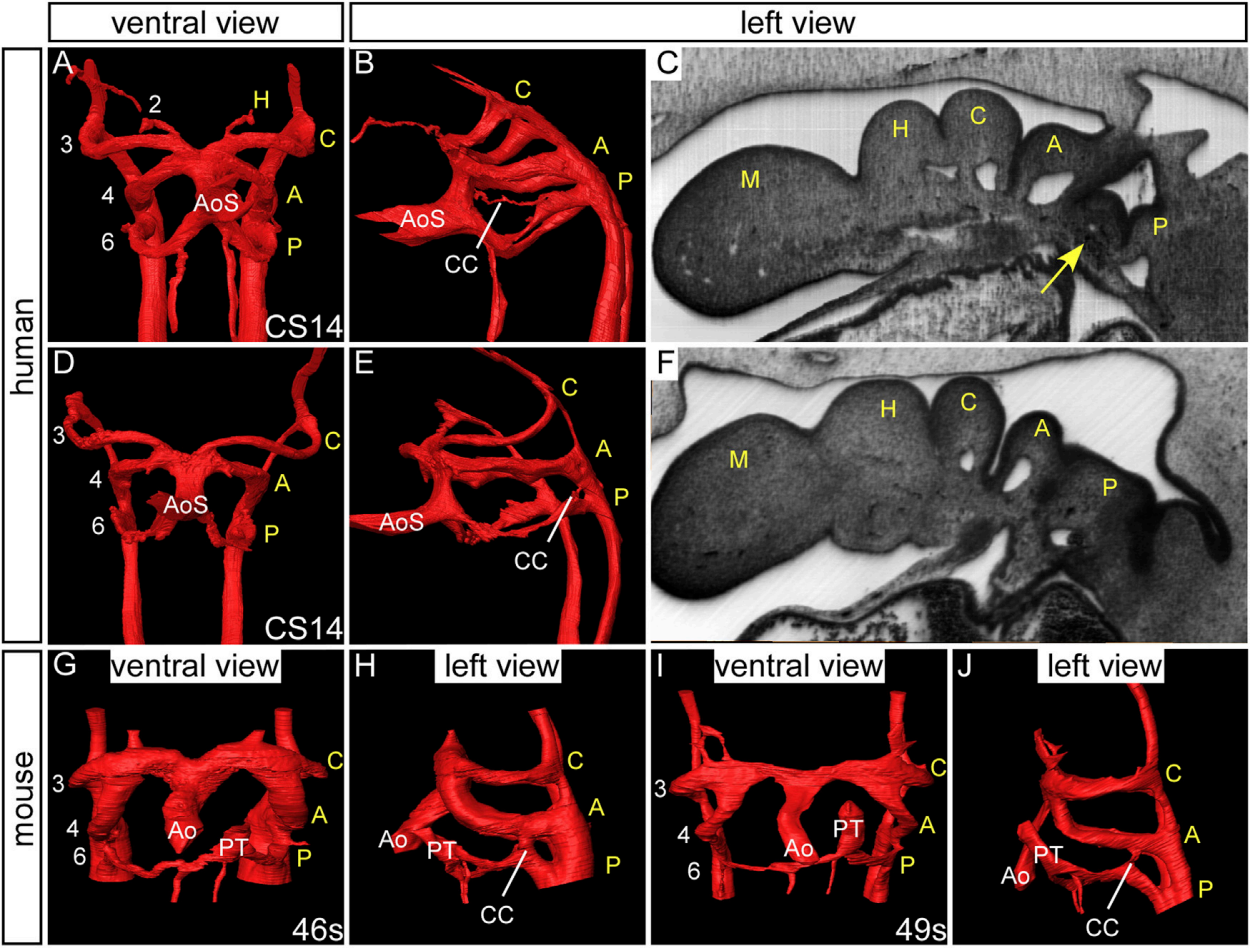
Collateral channels in human embryos. 3D reconstructions were made from high resolution episcopic microscopy datasets. Human embryos at CS14 have three pairs of symmetrical arch arteries and the aortic sac is unseptated **(A, D)**. A collateral channel is seen emanating from the dorsal surface of the artery of the pulmonary arch and almost connecting with the aortic sac **(B)**. The collateral vessel is contained within a block of mesenchyme (arrow, **(C)** which is not observed in the human embryo without this type of collateral channel **(F)**. Another type of collateral channel is observed connecting the pulmonary pharyngeal arch artery to the base of the aortic pharyngeal arch artery **(E)**. **(G–J)** Collateral channels connecting the pulmonary and aortic arch arteries are also observed in wild-type mouse embryos. Abbreviations: A, aortic pharyngeal arch artery; Ao, aorta; AoS, aortic sac; C, carotid pharyngeal arch artery; CC, collateral channel; H, hyoid pharyngeal arch artery; M, mandibular pharyngeal arch artery; PT, pulmonary trunk; P, pulmonary pharyngeal arch artery; s, somites. Adapted from (Bamforth et al., 2013).

Various studies were published at the beginning of the 20th century in different species proposing evidence for the presence of so-called “fifth arch arteries” (Lewis, 1906; Locy, 1907; Reinke, 1910). In an important study of human embryos, Tandler presented evidence of 13 examples of such “fifth aortic arch” arteries from the large collection kept in Vienna (Tandler, 1909). He described a consistent pattern, with the fourth pouch having a hook-like structure, which he considered to represent a fifth pharyngeal pouch. The additional tissue was named the ultimobranchial, or postbranchial, body. The rudimentary, and sometimes incomplete, alleged artery of this presumed fifth arch was always found in the furrow between the 4^th^ and the 5^th^ pouches, and was seen only in embryos between CS14 and 16. We also identified such an incomplete artery in an embryo at CS14. It was housed within a block of mesenchyme, and traversed from the dorsal extent of the artery of the pulmonary arch, almost connecting with the aortic sac (Figure 9) (Bamforth et al., 2013). We now interpret the channel to represent a collateral vessel and not a true fifth pharyngeal arch artery (Graham et al., 2019; Anderson et al., 2020).

Thus, although the reasoning of Boas has some merit in terms of evolutionary theory (Boas, 1887), the six-arch concept has never convinced all the experts. If a true arch artery is defined as connecting the aortic sac with the dorsal aorta, then none of the published examples satisfy the definition. For example, the images presented (Lewis, 1906; Reinke, 1910) show only a network of small vessels or a collection of small collateral channels.

It is frequent to find, however, collateral channels formed within the pharyngeal mesenchyme dorsally at the unions between the arteries of the aortic and pulmonary arches and the dorsal aorta. Such channels have been found in human embryos and up to half of developing wild-type mice (Lorandeau et al., 2011; Geyer and Weninger, 2012; Bamforth et al., 2013) (Figure 9) but were also identified at a higher incidence than controls in the Ts65Dn mouse model for Down syndrome (Lorandeau et al., 2011). Further evidence of mutant mouse models with fifth arch arteries is, however, lacking from the literature. It is the persistence of such collateral channels that provides good explanations for the lesions well described as double-barrelled aortas (Figure 6). The other lesions previously explained on the basis of persistence of the alleged arteries of a fifth set of arches within an overall complement of six are then better accounted for on the basis of remodelling of the aortic sac and its tributaries. Such remodelling explains the so-called bovine arch. This feature is relatively common and is found when both common carotid arteries arise from a single brachiocephalic trunk; it is usually asymptomatic (Aboulhoda et al., 2019). The pattern of branching of the aortic arch in cows, however, is very similar to that seen in humans, so the designation as “bovine arch” is very much a misnomer.

## Conclusion

The number of pharyngeal arches, and their associated arch arteries, have reduced in number throughout evolution. The jawed vertebrates develop six pharyngeal arch arteries but this is reduced to five in the air-breathing amniotes, with the ultimate pulmonary arch arteries functioning in the embryo as a right to left shunt to restrict movement of blood to the non-functioning lungs. The arteries of the pharyngeal arches are formed in a cranial-caudal symmetrical pattern in the embryo, and these remodel to form the typical asymmetric mature vessels in the amniotes. Should the arch arteries fail to form or remodel correctly, for example, through gene mutation, this can result in life-threatening congenital defects, which predominantly affect the adequate delivery of oxygenated blood in the neonate. We propose that names be given to the developing arches and their arteries, rather than numbers, particularly in humans as this is of greatest significance to those involved with understanding the anatomy of congenital heart disease. Biologists may of course continue to use the traditional numbering system. The naming system is designed for amniotes as it would be impossible to apply this to other clades that develop a different number of pharyngeal arches. As one could not simply transpose the names of the arches the numbering system would have to be used.
